# User-Driven Comments on a Facebook Advertisement Recruiting Canadian Parents in a Study on Immunization: Content Analysis

**DOI:** 10.2196/10090

**Published:** 2018-09-20

**Authors:** Jordan Lee Tustin, Natasha Sarah Crowcroft, Dionne Gesink, Ian Johnson, Jennifer Keelan, Barbara Lachapelle

**Affiliations:** 1 School of Occupational and Public Health Ryerson University Toronto, ON Canada; 2 Dalla Lana School of Public Health University of Toronto Toronto, ON Canada; 3 Public Health Ontario Toronto, ON Canada; 4 Laboratory Medicine and Pathobiology University of Toronto Toronto, ON Canada; 5 Department of Public Health Concordia University of Edmonton Edmonton, AB Canada; 6 Toronto Public Health Toronto, ON Canada

**Keywords:** Facebook, immunization, vaccination, antivaccination movement, social media

## Abstract

**Background:**

More people are searching for immunization information online and potentially being exposed to misinformation and antivaccination sentiment in content and discussions on social media platforms. As vaccination coverage rates remain suboptimal in several developed countries, and outbreaks of vaccine-preventable diseases become more prevalent, it is important that we build on previous research by analyzing themes in online vaccination discussions, including those that individuals may see without actively searching for information on immunization.

**Objective:**

The study aimed to explore the sentiments and themes behind an unsolicited debate on immunization in order to better inform public health interventions countering antivaccination sentiment.

**Methods:**

We analyzed and quantified 117 user-driven open-ended comments on immunization posted in the Comments section of a Facebook advertisement that targeted Canadian parents for recruitment into a larger study on immunization. Then, 2 raters coded all comments using content analysis.

**Results:**

Of 117 comments, 85 were posted by unique commentators, with most being female (65/85, 77%). The largest proportion of the immunization comments were positive (51/117, 43.6%), followed by negative (41/117, 35.0%), ambiguous (20/117, 17.1%), and hesitant (5/117, 4.3%). Inaccurate knowledge (27/130, 20.8%) and misperceptions of risk (23/130, 17.7%) were most prevalent in the 130 nonpositive comments. Other claims included distrust of pharmaceutical companies or government agencies (18/130, 13.8%), distrust of the health care system or providers (15/130, 11.5%), past negative experiences with vaccination or beliefs (10/130, 7.7%), and attitudes about health and prevention (10/130, 7.7%). Almost 40% (29/74, 39%) of the positive comments communicated the risks of not vaccinating, followed by judgments on the knowledge level of nonvaccinators (13/74, 18%). A total of 10 positive comments (10/74, 14%) specifically refuted the link between autism and vaccination.

**Conclusions:**

The presence of more than 100 unsolicited user-driven comments on a platform not intended for discussion, nor providing any information on immunization, illustrates the strong sentiments associated with immunization and the arbitrariness of the online platforms used for immunization debates. Health authorities should be more proactive in finding mechanisms to refute misinformation and misperceptions that are propagating uncontested online. Online debates and communications on immunization need to be identified by continuous monitoring in order for health authorities to understand the current themes and trends, and to engage in the discussion.

## Introduction

### The Role of the Internet in Vaccine Hesitancy

The World Health Organization (WHO) and its group of experts have identified vaccine hesitancy as an important issue facing immunization programs in the developed world [1]. This has been evident in Canada and other developed nations such as the United States and countries in Europe that have reported an increase in the number of outbreaks of vaccine-preventable diseases [2-7].

Many factors influence vaccine noncompliance or hesitancy; however, the role of the internet due to the abundance of online antivaccination sentiment and activists has been reported as an important concern [8-13]. A significant association was established between using the internet to search for vaccine information and negative parental perception of the risk of childhood immunizations [14]. More people are searching for health information online, including information on immunization [15,16]. Health professionals are concerned that parents seeking vaccine information online are being exposed to misinformation and antivaccination sentiment via websites and online communications on social media platforms [8,11,12,17]. Over the past decade, social media sites have gained popularity in Canada, where 67% of Canadian internet users are using social media on a daily basis [16], with most users being under the age of 35 years [18]. In Canada, Facebook is reported as the most popular social media platform, with usage rates higher than global and US averages [19,20]. Health information communicated in interactive platforms is of questionable accuracy, as it is often exchanged without the participation of health professionals or health organizations [17,21]. This exchange of misinformation online has the potential to influence parents’ decision to vaccinate their children [12,14,22,23] and may be contributing to suboptimal vaccination coverage among Canadian children [24] and increases in vaccine-preventable disease rates [25-28]. Results from the last Childhood National Immunization Coverage Survey show that 70% of Canadian parents surveyed reported being concerned about potential side effects of vaccines, and 37% believed that vaccines can cause disease [24]. A recent study by Dubé et al reported that vaccine experts perceive a decline in vaccination rates and that vaccine hesitancy is an important issue to address in Canada [29]. Furthermore, participants reported that dissemination of negative information online and lack of knowledge about vaccines were key issues in the causes of vaccine hesitancy in Canada [29].

Many studies have analyzed content from vaccine-critical websites and blogs found via search engines, as well as content posted on participative websites, chat rooms, and social media platforms such as Twitter, Facebook, YouTube, and Myspace [30]. These studies have identified similar themes, such as vaccine safety and effectiveness, alternative medicine, civil liberties, conspiracy theories, morality and misinformation, and mistrust of health professionals as the predominant arguments in the antivaccination movement [10,30,31]. Techniques such as skewing science, shifting hypotheses, and attacking critics have been reported as tactics of the online antivaccination community arguing against vaccination [11]. Themes underlying vaccine hesitancy can change over time and by place [13,29]; therefore, as coverage rates remain suboptimal in Canada and outbreaks of vaccine-preventable diseases become more prevalent, it is critical that we continue to build on previous research by analyzing themes in online vaccination discussions. Most research has focused on analyzing the content of discussions on sites or platforms that individuals would find via active research on immunization [30]. However, there is a gap in research in analyzing vaccine information that individuals may see without actively searching for information and could influence decisions on vaccination [30]. Ward et al proposed that future research on vaccine criticism on the internet should include analysis of more complex and interactive ways of information circulation, such as posts, likes, links, and retweets [30]. Furthermore, there is a need for more research to better understand vaccination sentiments specifically among Canadian parents.

**Figure 1:**
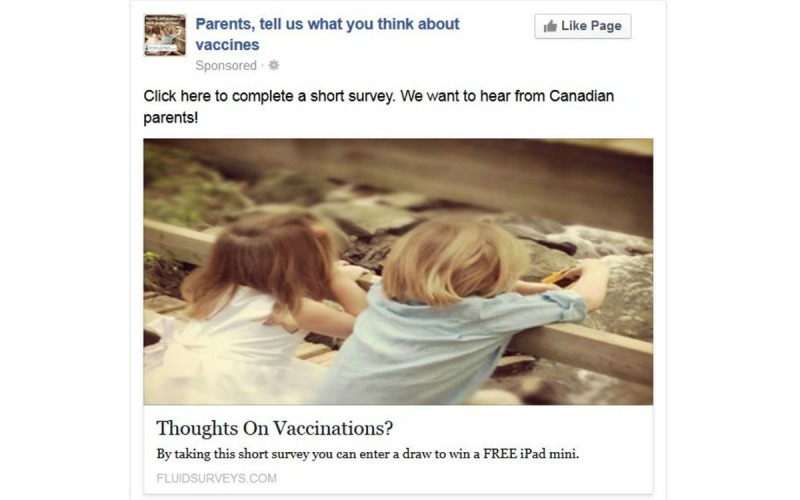
The most popular Facebook advertisement posted to Canadian parents’ News Feeds from December 12, 2013 to January 11, 2014.

**Figure 2:**
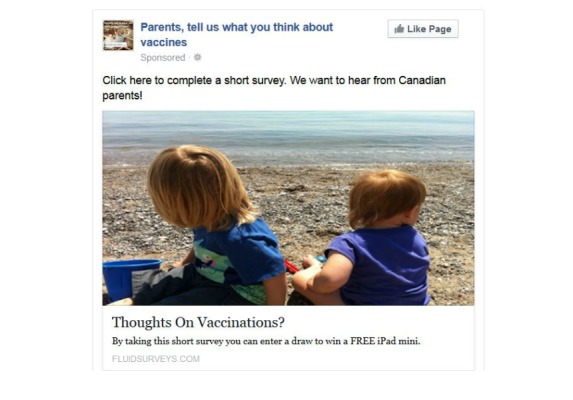
The second most popular Facebook advertisement posted to Canadian parents’ News Feeds from December 12, 2013 to January 11, 2014.

From December 12, 2013 to January 11, 2014, we posted 6 different Facebook advertisements linked to a Web-based survey on childhood immunizations to the Facebook News Feeds of Canadian parents as part of a larger research study [32]. The advertisements reached over 100,000 Canadian parents who matched the following inclusion criteria: (1) located in Canada, (2) 18 years of age or older, (3) parent of a child aged 0 to 15 years, and (4) displaying a profile in French or English. Overall, women represented the majority of Facebook users reached by the advertisements and who also clicked on the advertisement to the Web-based survey [32]. Two advertisements (Figure 1 and Figure 2) had the highest number of views from unique Facebook users reaching 74,572 users and 38,643 users, respectively, and the highest click-through rates to our online survey [32]. Further details on the methods and results of this recruitment strategy are available [32]. The advertisements did not provide any information on immunization, did not try to solicit discussion, and were not posted, shared, liked, or promoted by the researchers. The advertisements did not provide any information on immunization, did not try to solicit discussion, and were not posted, shared, liked, or promoted by the researchers. The Comments section of the advertisements was accessible, and this created an unsolicited and spontaneous discourse where users posted comments on immunization to the 2 most viewed advertisements (Figures 1 and 2).

### Objective

This study investigated a unique interactive debate on Facebook resulting from the above Facebook advertisements to recruit parents in immunization research. Our objective was to qualitatively analyze and quantify the content of users’ posts to describe the main vaccination sentiments and themes of an online immunization debate of Facebook users who commented on our posted advertisements, in order to better understand the vaccination debate and to identify underlying themes. We addressed this by asking 2 questions. First, what are the main vaccination sentiments (eg, anti- or provaccination) in the online debate? Second, what are the main themes on vaccination by type of sentiment? This study will add to the body of research on online vaccination discussions by analyzing a posting not intended for interaction that individuals could see without actively searching for information on immunization. The results will assist health professionals in understanding some of the content on vaccine information being shared online in order to help guide messaging and the development of online interventions.

## Methods

### Content Analysis

In this study, we qualitatively analyzed and quantified the content of open-ended comments posted by Facebook users. On January 11, 2014, at the end of the 4-week recruitment period, we captured and saved all user comments posted in the Comments section of the Facebook advertisements. We included all comments in French or English that contained any message on immunization. We excluded any comments that did not pertain to immunization (eg, comments on the advertisement itself, “lol”). We did not capture any identifying information from the Facebook users, and we removed the advertisement (along with the posted comments) from Facebook immediately at the end of the recruitment period; thus, no captured comments can be directly or indirectly linked to any Facebook user.

### Data Analysis

After comment capture, 2 raters (JLT and BL) independently coded the comments on the type of message, the sex of the user, the main message of the comment, and the claims made in the comment. To increase validity, the 2 raters independently categorized the comments and resolved any difference to reach 100% consensus based on discussion and a clear framework previously established [33-35]. A third rater was available if consensus was not attainable.

We measured user interaction by the number of “likes” for specific comments. Commentators either simply made comments or provided a link to vaccine information online. Thus, we classified the type of comment as comment only, comment with link to accurate information or trustworthy source, or comment with link to inaccurate information or nontrustworthy source. We classified trustworthy sources as links to government or reputable associations or scientists. We classified accurate information as websites with information or statistics from government sources or peer-reviewed studies. We classified remaining links as nontrustworthy or inaccurate. We determined the sex of the commentator by using the user’s name, photo, or comment and classified sex as not clear if one or both raters had any uncertainty.

We categorized the main message of the comments as positive, negative, hesitant, or ambiguous. We coded the comments as positive if the central message supported vaccination, portraying it positively (eg, describing the benefits or safety of vaccination, promoting vaccinations, describing the risks of not vaccinating or low risk of vaccinating) [36]. We coded comments as negative if the central message portrayed vaccination negatively (eg, emphasizing the risk of vaccination, opposing vaccination, promoting distrust in vaccine science, making allegations of conspiracy or collusion) [36]. If the central message portrayed indecision or uncertainty on the risks or benefits of vaccination (eg, questions or concerns about risk or safety, requests for information or links, questions regarding others’ decision to vaccinate), we coded the comments as hesitant. If the main message was not clear, we coded the comment as ambiguous. We then used two separate coding schemes to subcategorize the content: one for the negative, hesitant, and ambiguous comments and one for the positive comments.

We subcategorized the claims in the negative, hesitant, and ambiguous comments based on the themes of determinants of vaccine hesitancy suggested by the WHO’s Strategic Advisory Group of Experts Working Group (SAGE WG) on Immunization [37,38]. The SAGE WG matrix organizes vaccine sentiment into three domains: contextual influences, such as socioeconomic barriers, mistrust in the pharmaceutical industry, or religious values; individual and social group influences, such as personal knowledge or perceptions of risk; and vaccination and vaccination-specific issues, such as the vaccination schedule or characteristics of the vaccine; each main theme contains specific subcategories [37-39]. We categorized claims about vaccination within the comments according to the major themes and subthemes; claims could be classified into one or more themes and subcategories within the themes. We chose the SAGE WG matrix as the coding framework because it was developed by experts to include all known and potential determinants of vaccine hesitancy based on a thorough systematic review and expert opinion [37,38]. We created a category of other for any claim not covered by the SAGE WG matrix as determined by rater consensus [40]. The material was read several times prior to coding to ensure it fit the preconceived framework and to identify any other themes. Definitions of the framework categories were researched and discussed between the raters prior to coding. Both raters manually coded and discussed material from a random sample of respondents prior to independent coding.

The SAGE WG coding framework did not accurately capture the themes in the positive comments; thus, we categorized the claims in the positive comments based on broad themes in the data, with both raters independently generating categories and reaching consensus to develop the final coding scheme [33-35,40]. No new codes arose after approximately 40% of the comments were assessed.

The 2 raters independently categorized all comments (negative and positive) and claims within the comments, and achieved over 95% consensus. The raters met once to discuss items where consensus was not reached and achieved 100% consensus based on discussion and preestablished frameworks and criteria [33-35].

We conducted descriptive statistics to quantify respondent characteristics, main messages, and identified themes. Raters conducted content analysis with NVivo 10 qualitative data analysis software (QSR International) and quantified the analysis with descriptive statistics using Microsoft Office Excel 2007 (Microsoft Corporation). We obtained ethical approval from the University of Toronto’s Office of Research Ethics, Toronto, ON, Canada (REF#29309).

## Results

### Respondent Characteristics, Main Messages, and User Interaction

The advertisements generated 117 comments by 85 unique Facebook users after we excluded 9 comments not meeting the inclusion criteria. Of the 85 commentators, 77% (65/85) were female, 14% (12/85) were male, and for 9% (8/85) the sex was not clear. The majority of the comments were comments only (103/117, 88.0%), and 11.9% (14/117) posted links to websites. Of the 14 website links, 2 were from trustworthy sources, with 1 linked to a trustworthy source with accurate information (a government website with official statistics) and 1 linked to an online news story with accurate information posted from a government source. The main message of 43.6% (51/117) of comments was positive, followed by 35.0% (41/117) negative, 17.1% (20/117) ambiguous, and 4.3% (5/117) hesitant. Comments with the most interaction (20 or more likes) had mostly positive main messages (8/9, 89%) and 1 negative. The following 2 redacted positive comments had the most interaction (43 and 40 likes, respectively) and highlighted the predominant theme within the positive comments: the benefits of vaccines versus the risk for children and others in becoming infected with the disease (indicated as theme 1 in the comments below). In addition, the 2 other most identified themes were represented within these comments: parents who do not vaccinate their children are uneducated (theme 2), and vaccines do not cause autism (theme 3). Note that we redacted comments solely for the purpose of omitting words and sentences inconsequential to the context and analysis.

Vaccinating your children is the best way to prevent them (and others) from getting viruses and diseases...you are essentially protecting them from the awful signs and symptoms of the disease...the benefits out way the risks (Theme 1). Why do you think small pox was eradicated? Bc enough people around the world got the vaccine for it and it had no one to spread to, therefore: eradicated!!! There is NOT as many people unvaccinated as vaccinated, 80% of the population vaccinate their children...that # is decreasing bc of people’s lack of knowledge...Your not idiots for vaccinating your children you are just uneducated about biomedical facts! (Theme 2)

What about the infants and people who are immuno-compromised who CANT vaccinate? They depend on those people who CAN vaccinate to be protected and not spread these things!! (Theme 1) I have a child with autism, and do NOT believe vaccines have ANYTHING to do with it! That has been disproven! (Theme 3)

Lack of knowledge or awareness was the most prevalent theme in the negative comments, as suggested by the misinformation on immunity and transmission of disease contained within the following most liked (40 likes) negative redacted comment:

If their was a breakout of tuberculosis, polio...the vaccinated children would not be amune! If a vaccine protects you & your children, why...are all the vaccinated children catching it? There is absolutely no evidence that outbreaks start from unvaccinated people!...Every time there’s an outbreak there’s as many vaccinated as unvaccinated people catching the disease. There is absolutely no protection from a disease from taking a vaccine!

### Themes in the Negative, Hesitant, and Ambiguous Comments

In the 66 negative, hesitant, or ambiguous comments, 130 claims were made on factors affecting vaccination decisions. Individual and social group influence was the predominant theme in the claims within the posted comments (85/130, 65.4%). Within this theme, 20.8% (27/130) of the claims displayed lack of knowledge or awareness on immunization (including misinformation and the belief in their own research and knowledge), with the majority (22/27, 81%) providing inaccurate information or misperceptions on immunization and some explicitly stating their belief in the credibility or accuracy of their knowledge and research (5/27, 19%). Approximately 18% (23/130, 17.7%) of the claims revealed a low perception of the risk of disease and need for the vaccine or a high perception of risk of adverse events associated with vaccination. Table 1 displays the identified themes according to the WHO SAGE WG matrix on vaccine hesitancy.

### Themes in the Positive Comments

In the 51 positive comments (and 2 hesitant comments with positive claims), we identified 74 claims on factors affecting vaccination decisions. Within these comments, the majority (29/74, 39%) of the positive claims stated concerns over nonvaccinating parents putting their children and others at risk of disease and death or stated how the benefits outweigh the potential risks, followed by claims that nonvaccinating parents are uneducated, unintelligent, or selfish (13/74, 18%) (Table 2).

**Table 1:** Negative, hesitant, and ambivalent claims posted by Facebook users on Facebook advertisements categorized by themes (n=130).

Themes		n (%)	Examples of claims within comments
**Contextual influences**		19 (14.6)	
	Mistrust in pharmaceutical industry or government transparency	18 (13.8)	Pharma wanna make money...Bottom line is that vaccination is all about $$$$$... The chances of your child dying from these diseases is highly unlikely. There is SO much gov involvement...
	Religious values	1 (0.8)	I come from a Mennonite background where we were not vaccinated.
**Individual and group influences**		85 (65.4)	
	Lack of knowledge or awareness (misinformation and belief in own knowledge or research)	27 (20.8)	Lmao the courts admitted to vaccines causing autism...But they did it quietly! If I find the article I will post it on here...I do not vaccinate my children and never will...liquid mercury is metal you are injecting into your children... The argument that an epidemic would break out if children were not vaccinated is proven incorrect by every Amish/Mennonite community that is thriving today. Recent studies have shown startling evidence that links autism directly to vaccines along with decreased brain function. If you would like sources to this I can provide them. All sorts of diseases have been directly linked to vaccines including and especially autism...I hope wise people everywhere choose to educate themselves before making this decision. From my observations, limited as they are, the immunized ones tend to be the ones lacking basic immunity.
	Risk or benefit of vaccination (perceived, heuristic)	23 (17.7)	...so in my opinion he still would have a chance of getting these illnesses if I vaccinated him so I don’t see the point in giving him something that WILL harm him for a CHANCE that he might not get sick...There are some vaccinations that (my) children will not get (like chicken pox) as I think it is an unnecessary risk... There is absolutely no protection from a disease from taking a vaccine! But there are many people who die from vaccines every year! Don’t fool yourself. EVERY TIME you vaccinate there is a risk, even of death. It is up to you to decide if that risk is what is right for your child. For some children it might be worth it, but for other children it isn’t worth it...There are risks and there are children that are much better off without vaccines.
	Health system and providers (trust and personal experience)	15 (11.5)	Ask your doctor?! No Doctor is God. They are all trained to say the same thing. The truth is none of us know the truth. Any health care professional will side with pro vaccine idea. I will not vaccinate my son. Do you even know what your injecting in your kid?
	Beliefs and attitudes about health and prevention	10 (7.7)	My children have needed to see a doc approximately never in their lives. They are a testament to a holistic lifestyle and natural immunity. My observations of most kids that have been vaccinated is that they seem to be endlessly ill and have had multiple courses of antibiotics in their short lives!!
	Experience with past vaccination	10 (7.7)	My son had convulsions after getting vaccinated, that was 19 years ago and no vaccines again.
**Vaccination or vaccination-specific issues**		5 (3.8)	
	Role of health care professionals	3 (2.3)	...my paediatrician & general practitioner both disagree with vaccinating...
	Vaccination schedule	2 (1.5)	None of this 3 in 1...Dangerous injecting 2-4 shots in a kid at one time...
**Other**		21 (16.2)	
	Parents’ right to choose and not be judged	18 (13.8)	I think every parent has the right to chose what is best for their child. I don’t think it’s right for other parents or people to judge others for what they decide!!! I find it incredibly interesting that so many people are bothered by someone else’s choice to vaccinate or not vaccinate. If you get vaccinated, who cares if someone else doesn’t, it’s not your life....Everyone needs to take a chill pill... Defend your vaccines all you want but don’t call us idiots for not taking them!
	Requesting information or sources	3 (2.3)	Do you have any sources for your input?

**Table 2:** Positive claims posted by Facebook users on Facebook advertisements categorized by themes (n=74)^a,b^.

Themes	n (%)	Examples of claims within comments
Vaccines prevent disease risk or benefit	29 (39)	No vaccine is 100% but those vaccinated can fight the illness more effectively. Herd immunity only works when we vaccinate. I wonder if some peoples opinions would change if we lived in a country where vaccination was not common, and these diseases were common... Some parents have chosen to opt out and Polio, Whooping Cough and Diptheria are recurring. This puts us all at risk. The benefits outweigh the risks. We do not want these diseases to return with a vengeance! I personally could not live with myself if my child got very sick or died from a preventable disease to which we have access to free immunizations for...Now of course I vaccinated my kids because they can protect them from death...If they were bad...Or caused autism they would have been out of the market and not given by doctors don’t you think? I have 4 kids ranging from 18 to ten months. It’s worth the risk getting vaccinated. I’ve seen what whooping cough and polio do to people. I promise, those who’ve had polio will probably get their kids vaccinated.
Parents who do not vaccinate are uneducated or unintelligent	13 (18)	If you’re going to be an idiot and not immunize, at least make sure you’re a well educated idiot... Wow, it never ceases to amaze me how ignorant and just plain dumb some people are... It’s idiots who don’t vaccinate their kids that cause outbreaks...people think that they know more than the medical community. I find people who don’t vaccinate are some of the most uneducated nut jobs...
Follow the advice of health care providers and trustworthy sources	12 (16)	...get your information from reputable sites ie health canada or the cdc. Stay away from those “crunchy granola” opinion- based websites Research does not include google off siting an article you found on Facebook. These people don’t even know the definition of a peer reviewed research paper or study...and if you can’t tell the difference you should try and trust that the medical professionals who do know... ...everyone should read official statistics and not internet mumbo jumbo. The internet has so much bs that it can make anyone’s perception a reality... Yup our society rallies around a former porn star/actress looking to continue her 15 minutes of fame instead of putting our trust in our medical and science community...Sad state of society I’d say!
Vaccines do not cause autism	10 (14)	Jenny McCarthy made the Hollywood rounds stating her son got autism from his vaccines...Since then it has been proven her son doesn’t even have autism nor do vaccines cause autism... I have a child with autism, and do NOT believe vaccines have ANYTHING to do with it! That has been disproven! The jury is not out on autism. The verdict is no link...
I am provaccine or vaccinate	10 (14)	Be smart...Vaccinate Myself, I am a believer in vaccinations but that’s just what I believe is right for my kids...

^a^We included 2 hesitant comments with positive claims in the analysis.

^b^Total percentage does not equal 100% due to rounding.

## Discussion

### Principal Findings

The majority of comments were clearly pro- (51/117, 43.6%) or antivaccination (41/117, 35.0%) with few comments vocalizing vaccine hesitancy (4.3%). Themes in the online debate followed those identified in the literature and mostly captured in the SAGE WG framework [30,37]. As reported in other studies analyzing online vaccination messages [31,37,41], information in the negative comments was often inaccurate and the risks of immunization were misperceived. Mistrust in the pharmaceutical industry, the government, and health system was also a recurring theme in the online debate and previously identified as an important theme in studies analyzing vaccine-critical websites [10,21,30,31]. The right to choose without being judged was expressed within many negative comments yet not identified in the SAGE WG framework. This theme could have emerged in response to several judgments made within the positive comments on the level of intelligence or education of nonvaccinators. However, the theme of civil liberties or parents’ right to choose has been reported in previous studies analyzing vaccine opposition website content [10,30,31,41]. Slightly more positive comments were posted than negative or hesitant, and positive comments received the most interaction. Although the majority of the positive comments did not provide any links or obvious information from health authorities, there was encouragement to seek out trusted sources and people. No commentator self-identified as a health professional. The debate also highlighted the persistence of the myth linking vaccines to autism. Seeman et al [42] also reported this persistent inaccuracy on the safety of the measles-mumps-rubella (MMR) vaccine in an online survey of Canadian parents, and Nicholson and Leask [21] reported that one-third of the participants in an online MMR vaccine discussion forum were critical of the vaccine, with the risk of adverse effects and autism and concerns with vaccine ingredients as the major themes. Furthermore, a recent Canadian survey reported that 28% of adults reported to believe that there is or be uncertain about a link between vaccines and autism [43].

As we targeted the advertisements at Canadian parents, most of the commentators likely represented this demographic. Most of the commentators were female, but we expected this, as the Facebook campaign biased the advertisement reach toward a female population [32]. The 2 most popular advertisements reached over 100,000 Canadian parents on Facebook [32]; thus, the posted comments would have been visible to other targeted and potentially vaccine-hesitant Canadian parents who chose not to respond, as well as an unknown number of individuals not targeted by the campaign. These online debates should be of concern to public health authorities, as the spread of misinformation and misperceptions can reach large audiences with the potential to negatively influence vaccine-hesitant and provaccine individuals [22]. In addition, the analysis of the online debate revealed the lack of knowledge and spread of misinformation on a platform not intended to solicit discussion. The presence of public health authorities online is limited to top-down dissemination of information with limited engagement in online debates. This lack of public health involvement online could potentially enable the unabated spread of antivaccination sentiment and misinformation that potentially affect vaccination decisions among hesitant and provaccine parents.

Identified themes, such as the perceived risk of adverse events versus the risk of disease, and misinformation on autism and other disorders, immunity, and vaccine ingredients, could be addressed with more communication messages tailored to the issues in the online discussions. Although some antivaccination activists may never be swayed by evidence, it is important for health authorities to provide information to those with genuine concerns or questions, and engage in online debates rapidly in a nonjudgmental and transparent manner. Parents’ right to choose and not be judged was an important theme among the negative comments. The issue of freedom and individual rights versus the notion of social good is a fundamental ethical issue in immunization programs and needs to be given careful thought in our communications on issues such as mandatory vaccination and exemption rights. Passive interventions such as increasing knowledge or reminder recalls have been shown to be the least effective in addressing vaccine hesitancy [44], and there is a need for more dialogue-based approaches targeted to specific subpopulations with an intended focus on social networks [44]. In a recent randomized controlled trial, Glanz et al [45] found that Web-based information delivered on vaccines via social media platforms during pregnancy can have a positive impact on parental vaccine decisions. However, communication strategies on immunization via social media are still not well understood, and caution must be used to prevent legitimizing vaccine hesitancy [46]. Social media can be an important communication tool for public health; however, the content of online debates needs to be better monitored to identify the predominant themes, the type of misinformation, or specific requests for information, and to understand the determinants among Canadian parents [46,47]. This study adds to this body of research and highlights the major themes in one online debate, as well as the need for ongoing monitoring due to the extent of misinformation being shared.

Although online monitoring is essential, we need to better understand who should be engaging online to rebut misinformation and spread accurate and scientifically valid information on immunization. Mistrust in health care professionals and the government has been reported as an important determinant in vaccine hesitancy [30,37,48,49]; thus, alternative spokespeople (eg, influential mommy bloggers or celebrities) may need to be considered in the delivery of expert-based information. However, a recent survey of Canadian adults reported that the majority trust physicians and public health officials for timely and credible vaccine information, while popular celebrities were the least trusted [43]. Further research is needed to determine the extent of public health involvement, and what interventions or messaging and by whom would have the most impact online. MacDonald et al [50] reported that no simple strategy exists in overcoming vaccine hesitancy and that health care workers and immunization program managers need to “become adept at recognizing and tackling hesitancy in all of its incarnations.” This includes detecting vaccine hesitancy in populations and subgroups, having communication plans to address antivaccination misinformation, and actively supporting vaccine acceptors [50]. Online silence from public health authorities could give the impression of agreement with antivaccination information or sentiment [50]. Adversarial approaches could be counterproductive [51]; thus, public health departments need to be proactive in their social media strategies by promoting the safety of vaccines and addressing misinformation with targeted and tested interventions and messaging [13,17,50]. As such, it would also be useful to develop a common matrix that captures the arguments of those engaging in online discussions to influence nonvaccinators and vaccine-hesitant individuals (ie, provaccinators) and to further research their impact. Furthermore, health authorities and researchers should consider the ethical implications of nonengagement when using interactive online platforms for public health communications and interventions.

### Limitations

This study was limited in that the analysis was of one online debate and not necessarily representative of the main themes in all online immunization debates. Furthermore, the target audience was self-selected Canadian parents on an online social media platform, and we collected the presented data in 2013 and 2014. Thus, the results are not generalizable to a larger population, and the themes underlying vaccine hesitancy may have changed for this population, as they can be context specific, varying across time, place, or vaccine [13,37]. Thus, it is imperative that the online conversation be continually monitored in various subgroups and over time in order to identify current themes and trends to tailor public health communications on immunization to specific audiences. Although we did not intend the advertisement to elicit discussion on vaccines and clearly requested users to complete an online survey, it is possible that the advertisement unintentionally provoked discussion by asking for thoughts on vaccines. The type of messaging used should be considered when posting online advertisements, and the Comments section should be deactivated when appropriate and feasible. It is also important to note that we could have overestimated the total number of individual commentators (85 unique Facebook users), as it was not possible to verify whether the same individual had multiple accounts under different user names.

### Conclusion

The presence of over 100 comments posted on advertisements not intended as a discussion forum illustrates not only the strong sentiments associated with immunization but also the arbitrariness of platforms used for online debates. This unsolicited online debate is evidence of the importance of monitoring online discussions and of using technology capable of identifying immunization discussions among Canadian parents, as interactions are not just limited to vaccine-critical websites or groups and can occur via several platforms. The random nature of online debates will present a challenge for health authorities in terms of monitoring and engagement. Monitoring will need to include data mining with algorithms for keywords on immunization to quickly identify and engage in all public online communications on immunization. Health authorities need to identify methods to better leverage online platforms and networks in order to build trust, increase knowledge and access to information, and contest misinformation and misperceptions. It would also be important to consider appropriate jurisdictional responsibilities among health authorities for online surveillance and communications in immunization discussions.

## Abbreviations

MMR: measles-mumps-rubella
SAGE WG: Strategic Advisory Group of Experts Working Group
WHO: World Health Organization
